# Inhibiting Interleukin-19 Activity Ameliorates Esophageal Squamous Cell Carcinoma Progression

**DOI:** 10.1371/journal.pone.0075254

**Published:** 2013-10-09

**Authors:** Chung-Hsi Hsing, Franky Antonius Kwok, Hung-Chi Cheng, Chien-Feng Li, Ming-Shi Chang

**Affiliations:** 1 Department of Anesthesiology, Chi-Mei Medical Center, Tainan, Taiwan; 2 Department of Anesthesiology, College of Medicine, Taipei Medical University, Taipei, Taiwan; 3 Institute of Biochemistry and Molecular Biology, College of Medicine, National Cheng Kung University, Tainan, Taiwan; 4 Department of Pathology, Chi-Mei Medical Center, Tainan, Taiwan; Vanderbilt University, United States of America

## Abstract

**Background:**

IL-19 is expressed in esophageal squamous cell carcinoma (SCC), but its biological effect on esophageal cancer remains unclear. We determined the correlation between IL-19 expression levels and clinicopathological variables and explored the effects of IL-19 on the esophageal SCC *in vivo* and *in vitro.*

**Methodology/Principal Findings:**

We determined the expression levels of esophageal SCC tissues from 60 patients using immunohistochemistry. We examined the effects of IL-19 on intracellular signaling, cytokines production as well as proliferation, colonization, and migration in the human esophageal SCC cell line CE81T. Monoclonal antibodies (mAbs) against IL-19 (1BB1) and its receptor IL-20R1 (51D) were used to antagonize the effects of IL-19. We injected SCID mice with CE81T cells and then treated them with anti-IL-19 mAb or control IgG every 3 days and determined tumor growth for 32 days. Of the 60 esophageal SCC patients, 36 patients (60%) were IL-19 strongly stained, which was associated with advanced tumor stage. CE81T cells expressed IL-19 and its receptors. IL-19 induced phosphorylation of STAT3, P38, JNK, ERK1/2, Akt, and NF-κB in CE81T cells. IL-19 promoted the proliferation, colonization, and migration of CE81T cells, which were antagonized by 1BB1 and 51D. IL-19 also induced expression of the transcripts of TGF-β, cyclin B1, CXCR4, and MMP-1 in CE81T cells. In CE81T tumor-bearing mice, 1BB1 reduced tumor growth and downregulated TGF-β, cyclin B1, MMP-1, and CXCR4 expression in tumors.

**Conclusions/Significance:**

IL-19 affects the pathogenesis of esophageal cancer. IL-19 mAb (1BB1) is potentially a potent drug for esophageal cancer therapy.

## Introduction

Esophageal cancer is an important worldwide malignant disease with a high mortality rate [1]–[3]. The prognosis of esophageal cancer is poor because of extensive local invasion and frequent lymph node metastasis [4], [5]. Current studies report that much of the cancer progression is related to inflammatory cytokines, which affect cancer cell proliferation [6], inhibit apoptosis, induce pro-survival signals and angiogenesis [7], and promote tumor growth [6], [8]–[10]. They are probably involved in the mechanism tumor cells use to evade the immune surveillance system and in tumor microenvironments that affect the progression of cancer [6], [8]–[12].

A variety of direct cell-extracellular matrix (ECM) interactions are also involved in tumor progression and metastasis [13]. MMPs such as MMP-1 and MMP-2 were found to be important in proliferation, invasion, and migration of esophageal cancer cells [14]. In addition, chemokine receptor (CXCR4) was also associated with cancer cell survival, proliferation, chemotaxis, migration, and adhesion [15], and significantly correlated with lymph node metastasis in esophageal cancer [16].

IL-19 is a member of the IL-10 family cytokines (IL-10, -19, -20, -22, -24, -26, -28, and -29) [17]–[20]. IL-19 acts on multiple cell types by activating a heterodimer receptor complex of IL-20R1/IL-20R2 [21], [22]. IL-19 is upregulated by lipopolysaccharide (LPS), granulocyte-macrophage colony-stimulating factor (GM-CSF), IL-6, and tumor necrosis factor (TNF)-α [23], [24]. IL-19 also induces apoptosis in lung epithelial cells, stimulates liver cells to produce reactive oxygen species (ROS), and promotes neutrophil chemotaxis [25]. Clinically, IL-19 is induced in post-cardiopulmonary bypass inflammatory response and severe sepsis, which indicates that IL-19 may be involved in the pathogenesis of systemic inflammatory diseases [24], [25]. IL-19 is also involved in various inflammatory diseases such as psoriasis [26]–[28], asthma [29], and rheumatoid arthritis [30], [31].

We recently reported [32] that upregulated IL-19 in breast cancer promotes tumor progression and affects clinical outcome. We also found [33] that several types of tumor cells expressed IL-19, especially in squamous cell carcinoma of the skin, tongue, esophagus, and lung. IL-19 specifically activated an intracellular signal and induced proliferation of the cells, which indicated that IL-19 may act in an autocrine manner in oral cancer [33]. However, the role of IL-19 in esophageal cancer remains unclear. In this study, we investigated the effects of IL-19 in the pathogenesis of esophageal carcinoma *in vitro* and *in vivo*. We also determined the effect of anti-IL-19 monoclonal antibody (mAb) on reducing esophageal carcinoma tumor growth in a mouse model.

## Materials and Methods

### Patients and Tissue Specimens

This retrospective study was done in accordance with the guidelines of the Chi-Mei Medical Center Institutional Review Board (IRB9705-003). De-identified esophageal squamous cell carcinoma (SCC) samples of 60 patients obtained from the Pathology Archive, part of Chi-Mei Medical Center Tumor and Serum Bank between January 2009 and December 2011 were used for immunostaining. Healthy tissue samples were from non-pathological areas distant from tumors in surgical specimens (confirmed by histology examination). Non-tumor tissue samples with signs of inflammation were excluded [34]. The clinicopathologic variables evaluated are listed in Table 1.

**Table 1:** Associations between IL-19 expression in 60 esophageal SCC tumors with important clinicopathologic variables.

Parameter	Category	n	IL-19 expression			P value
			Low	High		
			24 (40%)	36 (60%)		
Primary tumor (T)	T1	2	2	0	0.019	
	T2	9	7	2		
	T3	33	11	22		
	T4	16	4	12		
Nodal status (N)	N0	14	10	4	0.034	
	N1	17	7	10		
	N2	16	4	12		
	N3	13	3	10		
DistantMetastasis (M)	M0	43	22	21	0.014	
	M1	17	2	15		
Stage	2	18	12	6	0.035	
	3	30	10	20		
	4	12	2	10		

### Cell Lines and Culture Conditions

Esophageal cancer cell line CE81T/VGH was purchased from The Food Industry Research and Development Institute (Hsinchu City, Taiwan) [35]. Cells were grown in Dulbecco’s modified Eagle’s minimal essential medium (DMEM; Gibco BRL, Gaithersburg, MD, USA) supplemented with 10% (v/v) fetal bovine serum (FBS; Gibco BRL), 100 U/ml penicillin, and 100 mg/ml streptomycin (Gibco BRL) and kept at 37°C in a 5% CO2/95% air atmosphere.

### Expression and Purification of hIL-19 and hIL-20R1 Recombinant Protein

A cDNA clone coded for the human (h)IL-19 and extracellular domain of hIL-20R1 sequences from was inserted into the expression vector of *Pichia pastoris* (pPICZ-α; Invitrogen, San Diego, CA, USA). We used affinity chromatography to express and purify hIL-19 and hIL-20R1 from the culture medium of the yeast cells [29]. The biological function was tested by treating IL-19 in peripheral mononuclear blood cells (PBMCs), which induced IL-10 production [36].

### Generating anti-hIL-19 and -hIL-20R1 Monoclonal Antibodies (mAb)

Monoclonal antibodies against hIL-19 (anti-hIL-19 mAb, 1BB1) and hIL-20R1 (anti-hIL-20R1 mAb, 51D) were generated following the standard protocols [29]. In brief, the hybridoma cells (1×10^6^) were injected intraperitoneally into pristine-pretreated BALB/c mice. Ascites fluid was collected after 2 weeks, and then 1BB1 or 51D mAb were purified with a Protein-A column (Pharmacia, Uppsala, Sweden). We previously reported [25], [33] that 1BB1 neutralized hIL-19. The 1BB1 mAb specifically recognized IL-19 but not other human IL-10 family cytokines such as IL-10, -20, -22, -24, and -26 [32].

### Immunohistochemistry

Paraffin-embedded-tissue samples were used for immunohistochemical staining with purified 1BB1 (diluted 1∶50) at 4°C overnight [27], [32], [33]. The pre-absorption test was done before the paraffin tissue sections had been incubated with recombinant IL-19 protein and 1BB1 (ratio, 10∶1). Incubating paraffin tissue sections with mouse IgG1 isotype (clone 11711; R&D Systems, Minneapolis, MN) instead of primary antibody was the negative control. Two investigators trained in pathology and blinded to the sample sources analyzed the histology and the IL-19 expression levels of at least five sections from each patient. The scoring of immunohistochemical stains in each specimen was determined using a histological score (H) [37] that was calculated using the following equation: H = Σ*Pi* (*i* +1), where *i* is the staining intensity of the stained tumor cells (0–4+), and *Pi* is the percentage (range: 0–100%) of stained tumor cells for each intensity. The IL-19 immunostaining was labeled low-grade (H<200) or high-grade (H≥200) as previous described [32].

### Immunocytochemistry

Anti-hIL-19 (1BB1), and anti-hIL-20R1 (51D) mAb were generated using standard protocols. Anti-hIL-20R2 mAb was purchased from Abcam, Cambridge, MA, USA). These three antibodies were used for immunocytochemical staining as previously described [38]. Briefly, cells were grown on sterile chamber slides, fixed and blocked, and then primary antibodies (anti-IL-19, -IL-20R1, or -IL-20R2 mAb) were added. After it had been incubated with secondary antibody, the immunoreactivity of the horseradish peroxidase-conjugated goat anti-mouse Ab (Santa Cruz Biotechnology, Santa Cruz, CA, USA) was detected using a substrate kit (DAB; Vector Laboratories, Burlingame, CA, USA). Incubation with nonspecific mouse IgG (R&D Systems, Minneapolis, MN, USA) as the primary antibody was the negative control.

### Reverse Transcriptase-polymerase Chain Reaction (RT-PCR)

Total RNA was extracted using TRIzol (Invitrogen, Carlsbad, CA, USA), and then total RNA underwent reverse transcription (SuperScript II Reverse Transcriptase; Invitrogen) according to the manufacturer’s instructions. IL-19, -20R1, and -20R2 mRNA was amplified using RT-PCR with specified gene-specific primers (Table 2). The RT-PCR products were visualized on 2% agarose gels containing ethidium bromide. β-actin was used as an internal control.

**Table 2:** Primer pairs used in this study.

Factor	Primer sequence (5′-3′)	Product size (bp)
IL-19	F-GGCAATGTCAGGAACAGAGG	
	R-AGCGGAATAAGACAGCCTGA	326
IL-20R1	F-TCTGGTATGTTTTGCCCGTA	
	R- GCCTGCGACTCCAATAATGT	549
IL-20R2	F-GAAGTGGCCATTCTGCCTGCC	
	R-GGGAATGGCCTCTCCTTGCAC	300
TGF-β	F-CGGCAGCTGTACATTGACTT	
	R-TCAGCTGCACTTGCAGGAG	287
MMP-1	F- ATCCCTTCTACCCGGAAGTT	
	R- GTATTGTCTTGTTCCATGAAAG	418
CXCR4	F-TAAGCTGTCACACTCCAAGG	
	R-TCCATGAGCAGAGGCTCC	301
Cyclin B1	F- CCAGAAATTGGTGACTTTGCT	
	R- TGAACTAGTGCAGAATTCAGC	481
β-actin	F-GCTGGAAGGTGGACAGCGAG	
	R-TGGCATCGTGATGGACTCCG	520

### ELISA

Concentrations of IL-19 in cultured supernatants of CE81T cells were determined using ELISA with pairs of specific monoclonal or polyclonal antibodies as previously described [29], [39]. Results were expressed as the means of duplicate assays.

### Cell Proliferation Assay

CE81T cells were seeded at 3×10^4^ cells/ml in 24-well dishes and allowed to attach for 8 h, cultured in growth medium without fetal bovine serum (FBS) for 16 h, and then exposed to IL-19 at the indicated concentrations for 48 h. Cell proliferation was assessed using BrdU incorporation (BrdU ELISA colorimetric assay; Roche, Indianapolis, IN). To demonstrate the specific activity of hIL-19, 1BB1 or 51D mAb at a concentration of 10∶1 (mAb:IL-19) was added with IL-19, and the proliferation of the CE81T cells was monitored. All experiments were done in triplicate.

### Soft Agar Colony-forming Assay

Cells exhibiting exponential growth were suspended in complete growth medium containing 0.33% Bacto-agar (A-6013 Type 1 Low EEO; Sigma-Aldrich) and overlaid on 0.5% agarose gel in 30-mm dishes (10^4^ cells/dish). Medium containing IL-19 (200 ng/mL) was overlaid on the top agar. The dishes were maintained at 37°C in a humidified incubator (5% CO_2_, 95% O_2_) for two weeks. During this period, the medium was changed every 3 days. The number of visible colonies (>50 µm) were counted under a microscope. All experiments were done in triplicate.

### Real-time Migration Assays

CE81T cells were seeded at 1×10^5^ cells/ml in 6-well dishes and allowed to attach for 18 h. The cells were then exposed to medium containing hIL-19 (200 ng/ml). Cell migration kinetics was recorded using a JuLI Smart fluorescent cell analyzer instrument (JuLI Smart; Montreal Biotechnologies Inc. (MBI), Dorval, PQ, Canada) for approximately 12 h. The result then analyzed using ImageJ Software (http://rsbweb.nih.gov/ij/). FBS (10%) was used as the positive control.

### Western Blotting

CE81T cells (1×10^6^) were plated in 6-cm dishes, starved for 16 h, and then stimulated with IL-19 (200 ng/ml) for the indicated times. Total cell lysate was collect by adding 1× RIPA buffer containing phenylmethanesulfonyl fluoride (PSMF) (RIPA:PSMF = 10∶1) and centrifuged at 13000 rpm at 4°C to collect the supernatant. Western blotting with antibody specific for phosphor-STAT3, phosphor-P-38, phosphor-Jun N-terminal protein kinase (JNK), phosphor-extracellular signal-regulated kinase (ERK), phosphor-nuclear factor-kappa B (NF-κB**)**, and phosphor-Akt and β-actin (Cell Signaling Technology, Beverly, MA, USA) was used following the manufacturer’s instructions. β-actin was used as an internal control.

### Real-time Quantitative Polymerase Chain Reaction (Real-time QPCR)

CE81T cells were stimulated with hIL-19 (200 ng/ml) for the indicated time. Total RNA was extracted as described above. The amplified template was detected using Maxima® SYBR Green/ROX qPCR Master Mix (2X) (Fermentas, Burlington, Ontario, Canada) with a real-time PCR system (LightCycler® 480; Roche, Basel, Switzerland) with gene-specific primer (Table 2). β-actin was used as an internal control.

### Tumorigenicity in BALB/c Scid Mice

Eight-week-old male BALB/c Scid mice were acquired from the NCKU Laboratory Animal Center. The mice were housed under strict pathogen-free conditions and given sterile food and water. The Committee on Animal Research at NCKU approved all procedures (No. 099072). *In vitro* cultured CE81T cells (1×10^6^ cells/0.2 ml DMEM with 10% v/v FBS) (Gibco BRL) were subcutaneously injected into the abdominal region of each mouse. Tumor development was assessed every 2 days one week after the injection of tumor cells. Thirty-two days after the injection, the mice were killed and their tumors were harvested for further measurement.

### Statistical Analysis

Statistical analysis was done using SPSS 14.0 (SPSS Inc., Chicago, IL). A χ^2^ test, Fisher’s exact test, Student’s *t* test, or Kruskal-Wallis one-way analysis of variance (ANOVA) test, as indicated, and then Dunn’s test, were used. Data are means ± standard deviation (SD). Significance was set at P<0.05.

## Results

### IL-19 Expression in Tumor Tissue was Correlated with Tumor Metastasis and Clinical Staging

**Figure 1:**
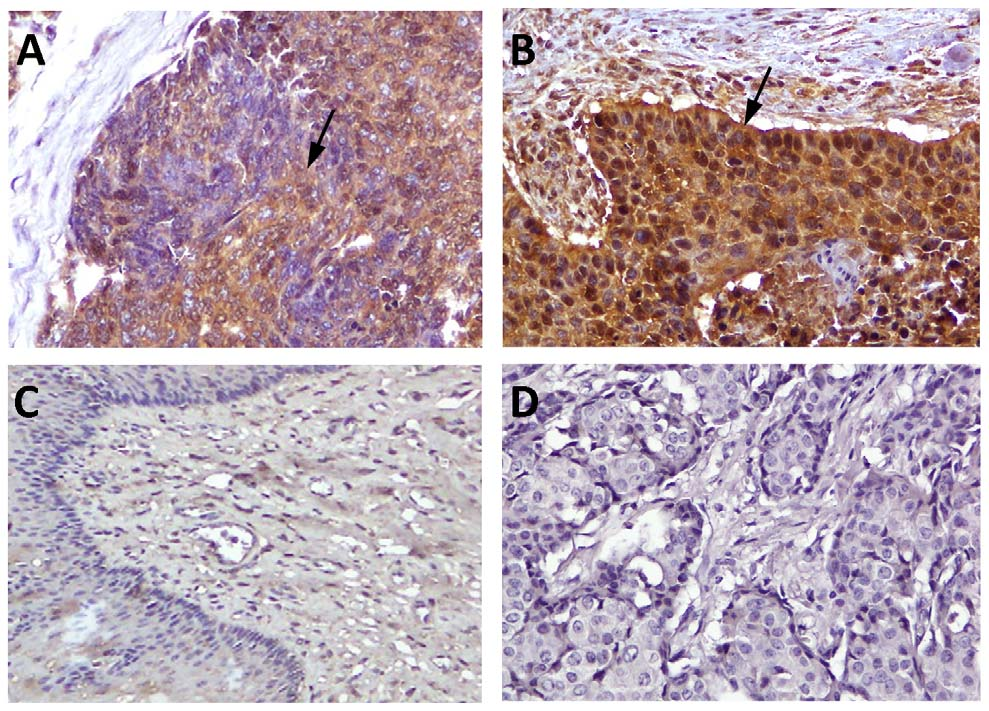
Immunostaining of IL-19 in esophageal cancer cells. Immunohistochemical staining showed that IL-19 staining intensity was high grade (H≥200) (A) or low-grade (H<200) (B) stained in esophageal SCC cells (arrows) but non/weakly stained in healthy esophageal epithelial cells (C) (magnification, ×400). (D) Mouse IgG was used as a negative control.

Sixty SCC of esophagus tissue samples were immunohistochemically (IHC) stained with 1BB1. Staining intensity was high-grade in 36 samples (Fig. 1A) and low-grade in 24 (Fig. 1B). Healthy esophageal tissue samples were non/weakly stained (Fig. 1C). High IL-19 expression was associated with advanced tumor stage and a high incidence of lymph-node metastasis and distant metastasis (Table 1). A strong correlation was substantiated between IL-19 expression with primary tumor status (T) (P = 0.019), nodal status (P = 0.034), distant metastasis (P = 0.014) and high tumor stage (P = 0.035). The findings of strong associations between IL-19 expression and several adverse clinicopathologic prognosticators suggested its crucial role in tumor progression of esophageal cancer.

### Esophageal Cancer Cells Expressed IL-19 and its Receptor IL-20R1/IL-20R2

**Figure 2:**
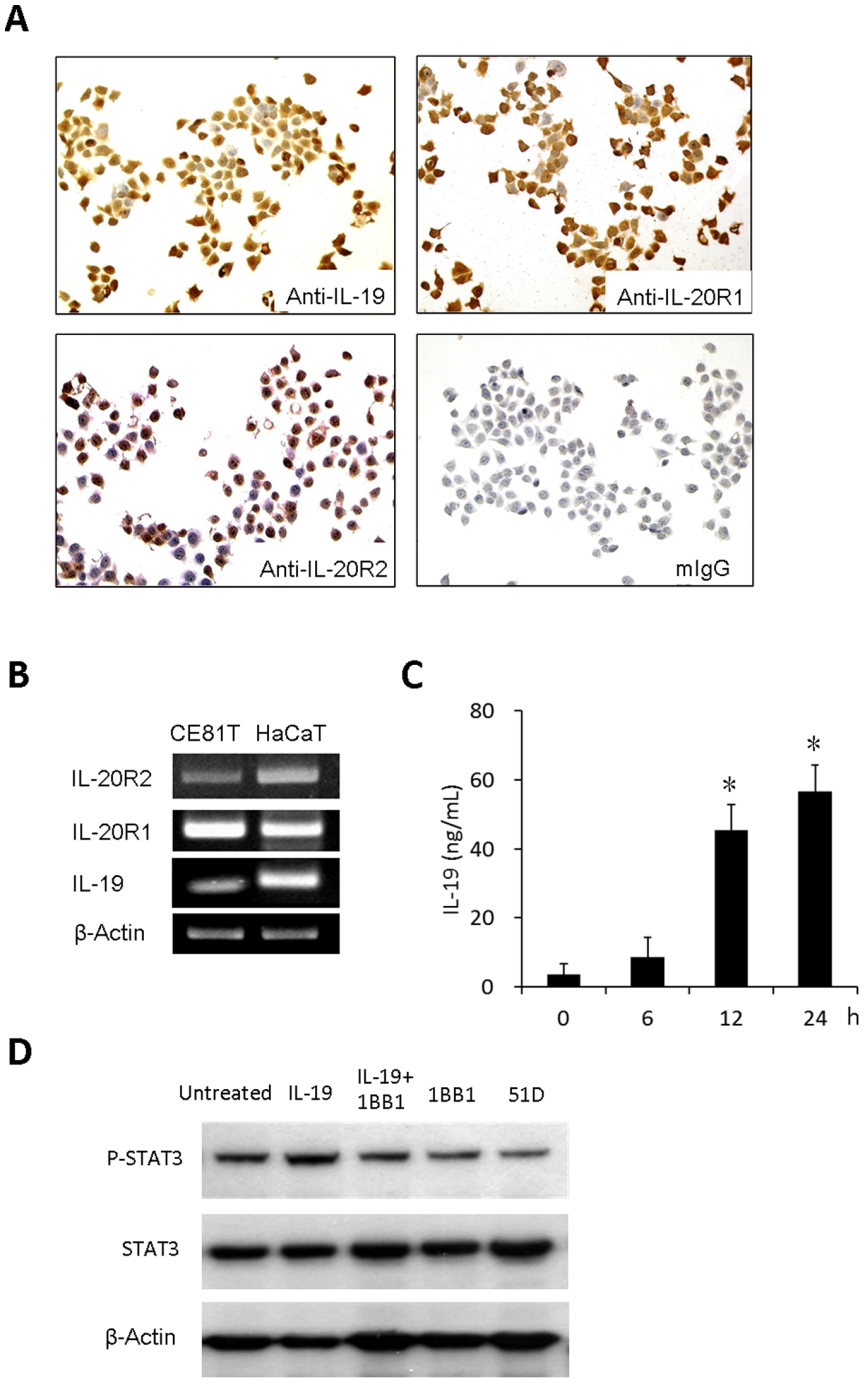
CE81T cells expressed IL-19 and its receptors IL20-R1 and IL20-R2 and IL-19 plays as an autocrine. (A) Immunocytochemical staining (mIgG was the negative control) and (B) real-time polymerase chain reaction analysis (HaCaT cells were the positive controls) of the effect of IL-19 and its receptors IL-20R1 and IL-20R2 on CE81T cells. (C) Concentrations of IL-19 in cultured supernatants of CE81T cells were determined at indicated times using ELISA. Data are mean ± SD. All groups: n = 3. *P<0.05 compared with 0 h group. (D) IL-19 increased STAT-3 phosphorylation in CE81T cells, which was attenuated by anti -IL-19 mAb (1BB1). 1BB1 and anti-IL-20R1 mAb (51D) also reduced endogenous STAT-3 phosphorylation in CE81T cells. All experiments were done 3 times and yielded similar results. Data are from a representative experiment.

To investigate the role of IL-19 in the pathogenesis of esophageal cancer, we first determined the expression of protein and mRNA of IL-19 and its receptors IL-20R1/IL-20R2 in esophageal cancer cell CE81T using immunocytochemical stains and RT-PCR, respectively. Both IL-19 and its receptors were expressed in CE81T cells (Fig. 2, A and B). We further determined the levels of secreted IL-19 by CD81T cells. After 12 h starvation, CD81T cells were incubated in cultured medium containing 10% FBS and then determined the levels of IL-19 in cultured medium at indicated times using ELISA. The concentrations of IL-19 were higher at 12 h than at 0 h (Fig. 2C).

To investigate the autocrine fashion of IL-19, we determined IL-19-induced intracellular STAT-3 phosphorylation using Western blotting. IL-19 (200 ng/mL) increased STAT-3 phosphorylation in CE81T cells after 12 h treatment which was attenuated by anti-IL-19 mAb (1BB1) (Fig. 2D). Furthermore, 1BB1 and anti-IL-20R1 mAb (51D) treatment alone also reduced CE81T cells STAT-3 phosphorylation (Fig. 2D), indicated that endogenous IL-19-induced STAT-3 phosphorylation was attenuated by 1BB1 and 51D.

### IL-19 Induced Cell Proliferation and Migration in Esophageal Cancer Cells

**Figure 3:**
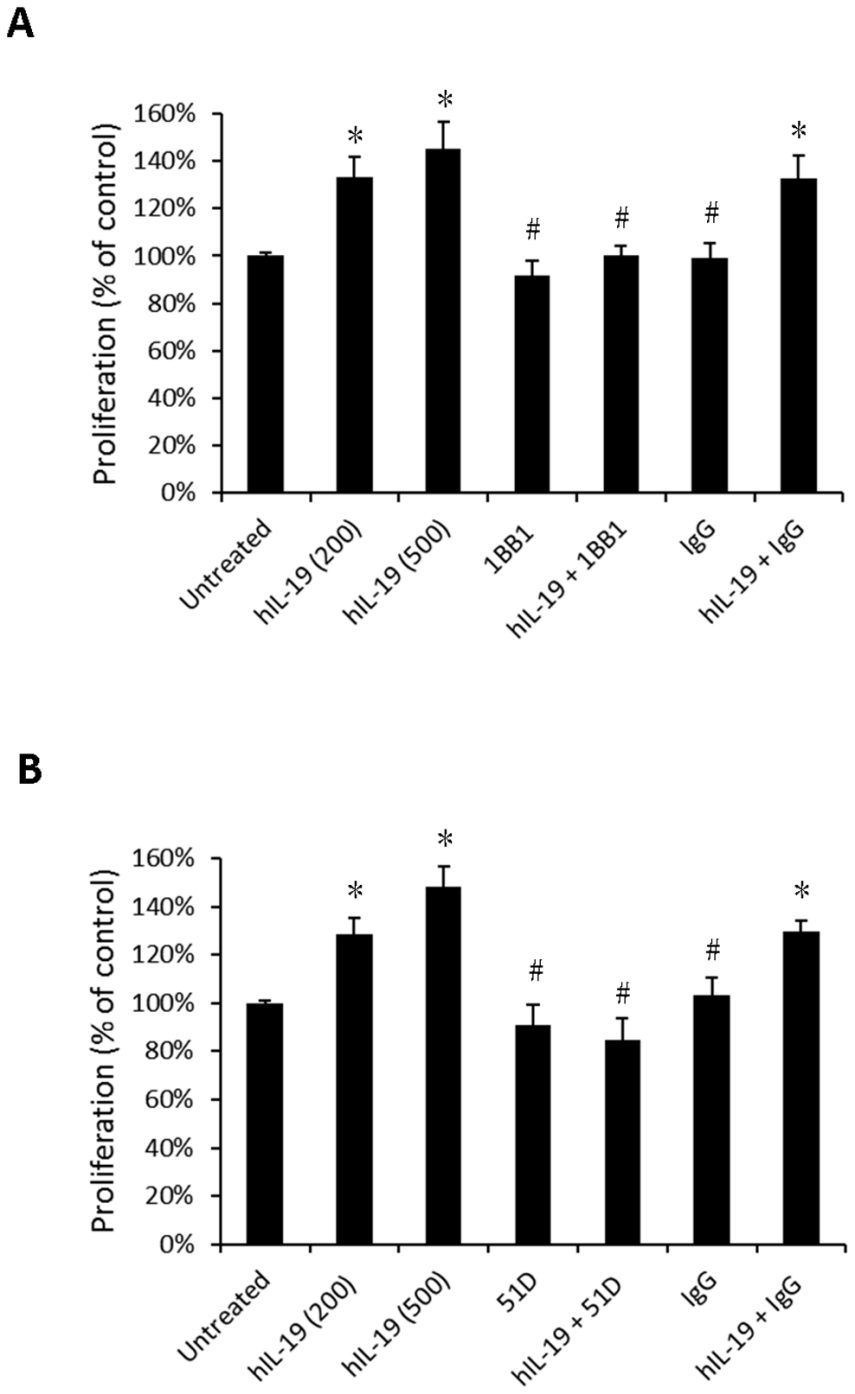
IL-19 induced cell proliferation in CE81T cells. (A) CE81T cells treated with human IL-19 (hIL-19) (200 ng/mL or 500 ng/mL, as indicated) and combined with hIL-19 monoclonal antibody (1BB1, 2 ug/mL). (B) CE81T cells treated with hIL-19 (200 ng/ml or 500 ng/mL, as indicated), and combined with anti-IL-20R1 monoclonal antibodies (51D, 2 ug/mL). Proliferation was analyzed using BrdU incorporation assays. IgG was the negative control. Data are mean ± SD. All groups: n = 3. *P<0.05 compared with untreated group, #P<0.05 compared with IL-19 group.

**Figure 4:**
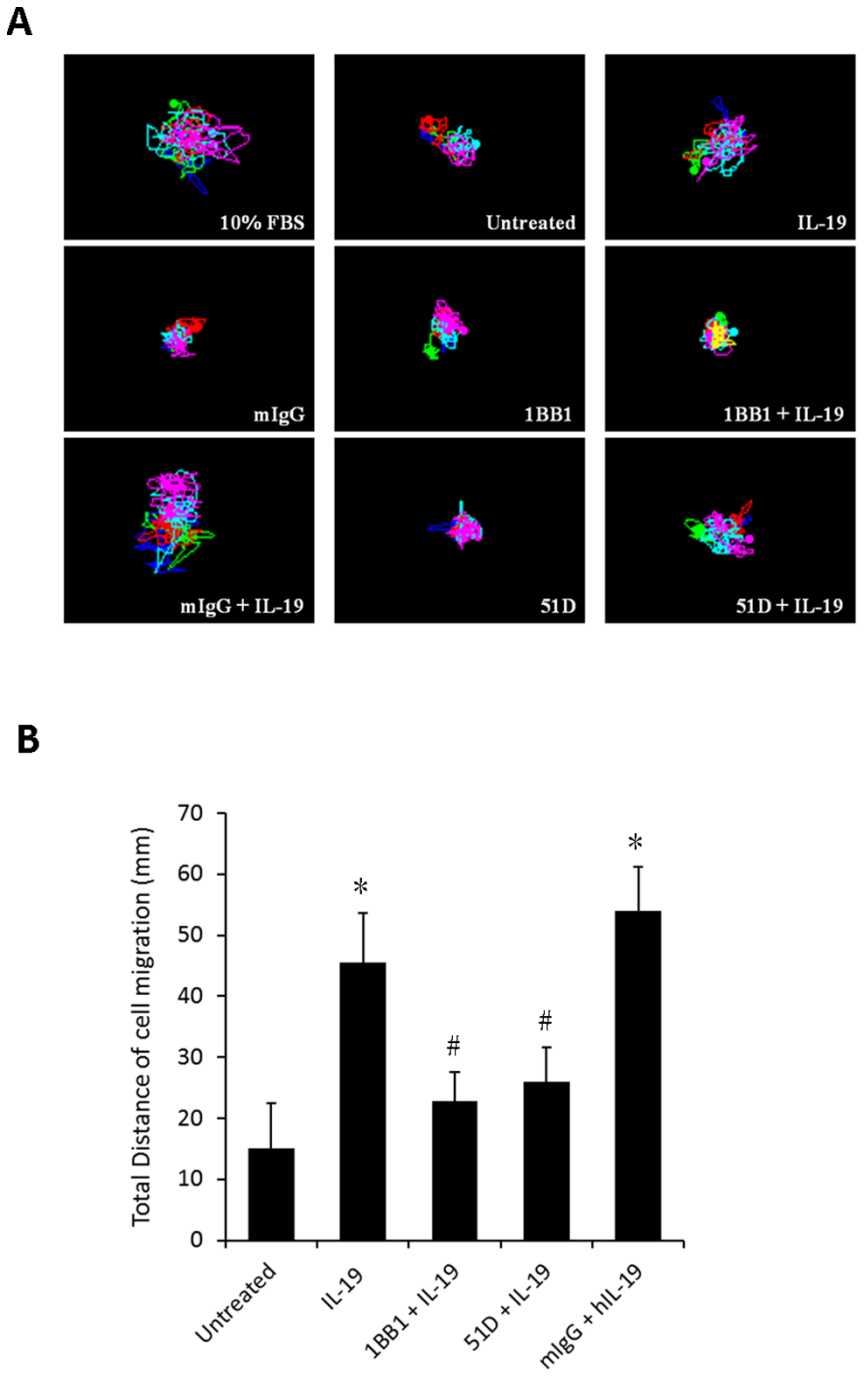
IL-19 induced migration in CE81T cells. Real-time migration for CE81T cells was monitored using a JuLI smart fluorescent cell analyzer. (A) Motion graph of CE81T cells (count = 5 cells). (B) Quantization of the motion distance (in µm) of CE81T cells (count = 5 cells) (10% FBS was the positive control; IgG, the negative control of anti-IL-19 monoclonal antibody (1BB1) and anti-IL-20R1 monoclonal antibody (51D). All groups: n = 3. *P<0.05 compared with untreated group, #P<0.05 compared with IL-19 group.

We next used BrdU incorporation assay to determine the effect of IL-19 on the proliferation of CE81T cells. IL-19 significantly induced CE81T cell proliferation, which was inhibited by anti-IL-19 mAb (1BB1) (Fig. 3A) and anti-IL-20R1 mAb (51D) (Fig. 3B). IL-19 specifically modulated esophageal cancer cell proliferation via its receptors. Tumor progression is usually characterized by migration and metastasis. To determine whether IL-19 affected cell migration in CE81T cell, a real-time migration assay was used to evaluate the migration ability of CE81T cells. IL-19 significantly promoted CE81T cell migration and anti-IL-19 mAb (1BB1) and anti-IL-20R1 mAb (51D) specifically inhibited the activity (Fig. 4).

### IL-19 Increased Colony Formation in Esophageal Cancer Cells

**Figure 5:**
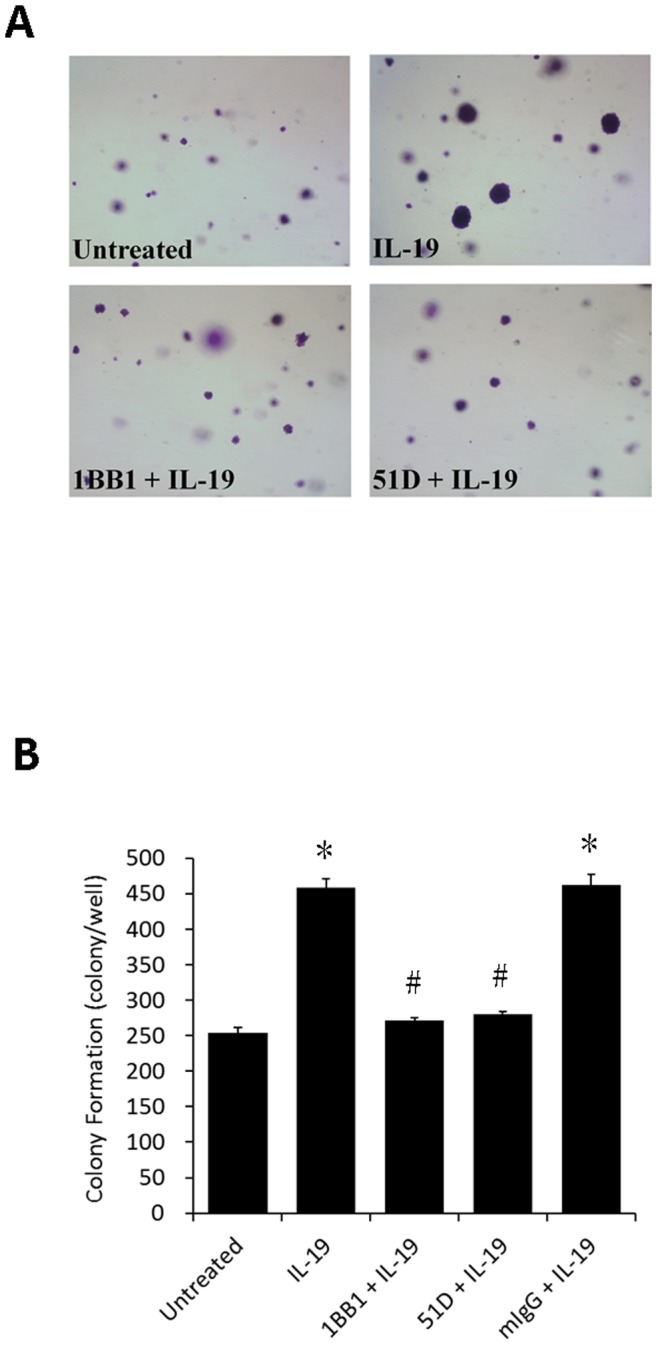
IL-19 increased colony formation in CE81T cells. Soft agar colony formation for CE81T cells treated with human IL-19 (hIL-19) (200 ng/ml) or hIL-19 combined with anti-IL-19 mAb (1BB1) and anti-IL-20R1 mAb (51D). (A) Photomicrographs of colonies were taken two weeks after plating. Representative data from three independent experiments are showed. (B) Quantitative analysis of colony formation. IgG was the negative control. All groups: n = 3. Data are mean±SD. *P<0.05 compared with untreated group, #P<0.05 compared with IL-19 group.

The initial step of local invasion of esophageal cancer was characterized by the increase of colony formation of the cancer cells. The colony formation was significantly higher in IL-19 treated CE81T cells than in untreated control, the activity of which was attenuated by 1BB1 and 51D mAbs (Fig. 5).

### IL-19 Induced Signal Transduction in Esophageal Cancer Cells

**Figure 6:**
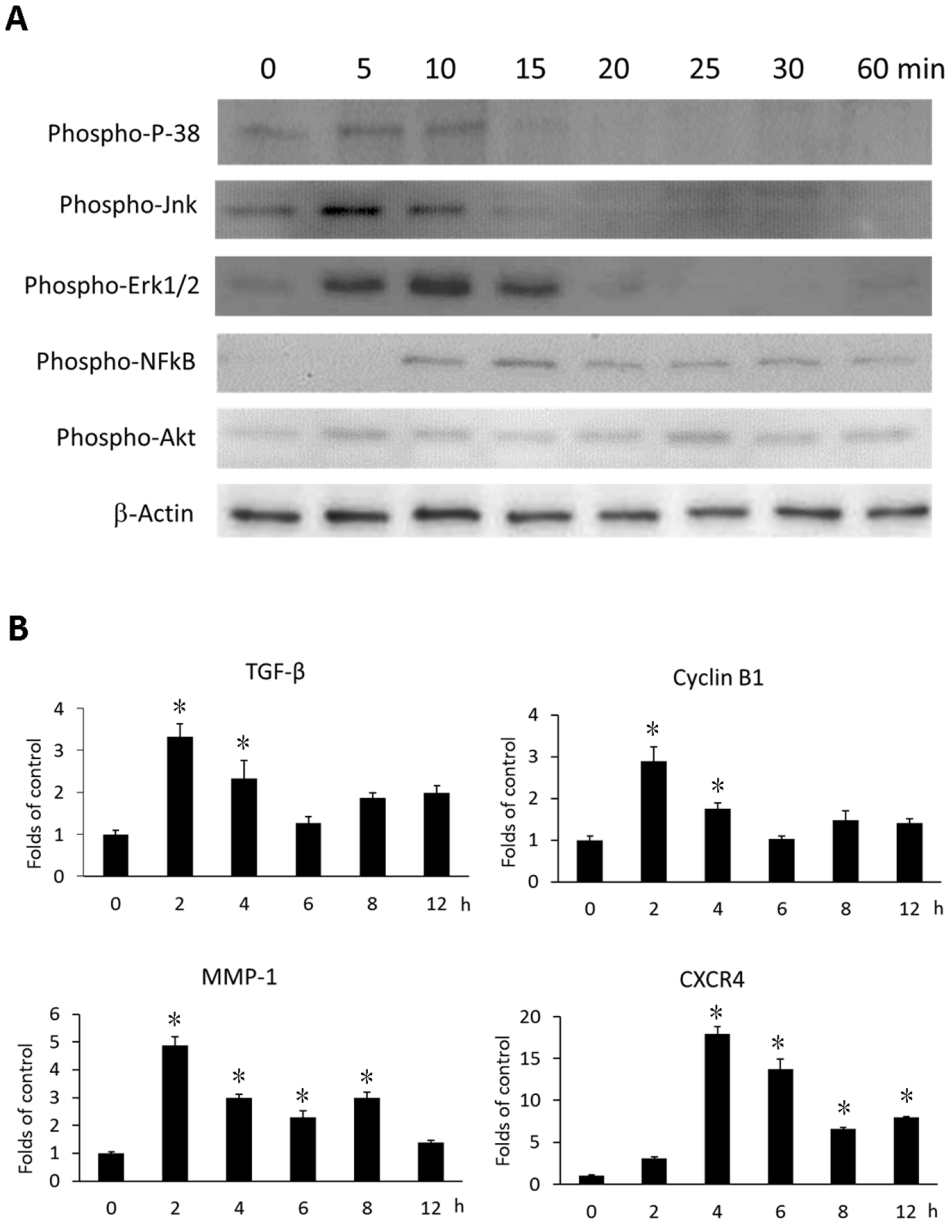
IL-19 activated intracellular signaling and induced cytokines expression in CE81T cells. (A) CE81T cells were treated with IL-19 (200 ng/ml) for 0, 5, 10, 15, 20, 25, 30, or 60 min and phosphorylation of P-38, Jnk, Erk 1/2, AKT, and NF-κB were analyzed using western blotting. β-Actin was the internal control. Data are representative of 3 independent experiments. (B) CE81T cells were treated with human IL-19 (200 ng/ml) for 2, 4, 6, 8, or 12 h. Expression of TGF-β, cyclin B1, MMP-1, and CXCR4 mRNA was determined using real-time qPCR. All groups: n = 3. Data are mean±SD. *P<0.05 compared with untreated (0 h) group.

Intracellular signal transduction plays a role in esophageal cancer [40] [41], we analyzed the effect of IL-19 on intracellular signaling in CE81T cells. IL-19 induced phosphorylation of P-38, Jnk, Erk1/2, Akt, and NF-κB in CE81T cells (Fig. 6A). These result suggested that IL-19 had triggered proliferation associated signals, and acted as a promoting factor for tumor growth.

### IL-19 Induced TGF-β, Cyclin B1, MMP-1, and CXCR4 Expression in Esophageal Cancer Cells

In esophageal cancer, TGF-β, MMP-1, and CXCR4 were upregulated and associated with cancer progression [14]. Cyclin B1 was also related to the proliferation of esophageal cancer cells [42]. We therefore treated CE81T cells with IL-19 for the indicated time and determined the expression of TGF-β, cyclin B1, MMP-1, and CXCR4 mRNA using real-time PCR. IL-19 upregulated TGF-β, cyclin B1, MMP-1, and CXCR4 mRNA (Fig. 6B). These results indicate that IL-19 affects the tumor invasion-associated factors in esophageal cancer progression.

### Anti-IL-19 Monoclonal Antibody Suppressed Tumor Growth and Expression of IL-19, TGF-β, MMP-1, CXCR4, and Cyclin B1 in Esophageal Cancer Cells *in vivo*

**Figure 7:**
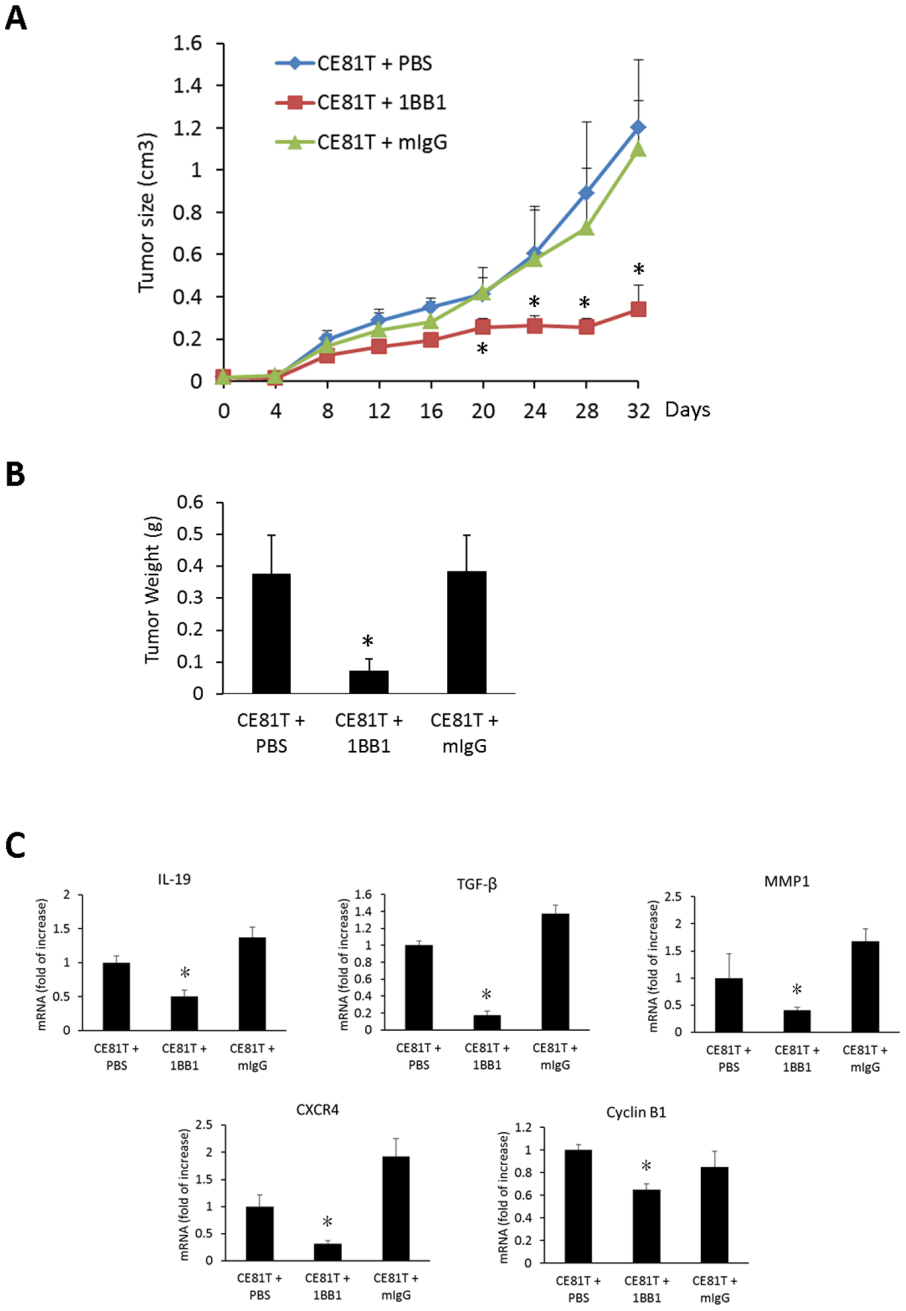
Anti-IL-19 monoclonal antibody treatment suppressed CE81T tumor growth *in vivo*. CE81T cells (1×10^6^) were subcutaneously injected into left flank of the mice. All mice were subcutaneously injected with anti-IL-19 mAb (1BB1, 15 mg/kg) or mIgG (15 mg/kg) or PBS as indicated every 3 days. (A) Tumor growth was determined every other day for 32 days. (B) After 32 days of CE81T cells injection, the mice were sacrificed and the tumors were harvested and weighted. Tumor weight of the 1BB1-treated group was less than that of PBS and mIgG-treated mice group. (C) Expression of IL-19, TGF-β, MMP-1, CXCR4, and cyclin B1 in CE81T tumors were determined using real-time qPCR. All group n = 6. Data are mean±SD. *P<0.05 compared with PBS and mIgG groups.

We next investigated the effect of 1BB1 on inhibiting of CE81T tumor growth *in vivo*. We subcutaneously (s.c.) injected CE81T cells into BALB/C SCID mice, treated the mice with a 1BB1 injection (15 mg/kg, s.c.) every 3 days, and then determined tumor growth for 32 days. Tumor size in the 1BB1-treated group was smaller than in the mIgG- and PBS-treated control groups (Fig. 7A). After 32 days, the mice were sacrificed and their tumors were weighed. Tumors in the 1BB1-treated group weighed less than in the mIgG- and PBS- treated groups (Fig. 7B). Moreover, IL-19 expression in the 1BB1-treated group was significantly lower than in the mIgG- and PBS-treated Control groups (Fig. 7C). In addition, 1BB1 treatment reduced the expression of TGF-β, MMP-1, CXCR4, and cyclin B1mRNA in tumors (Fig. 7C).

## Discussion

Inflammatory mediators in tumor microenvironments affect cancer progression [6], [8]–[10]. The present study provides evidence that IL-19 is associated with the pathogenesis of esophageal cancer. IL-19 expression in esophageal SCC is associated with tumor metastasis and clinical stage. Esophageal cancer cells expressed IL-19 receptors, which indicates that IL-19 is an autocrine factor in esophageal cancer. IL-19 directly affected esophageal cancer cell proliferation and migration and promoted tumor progression. It also induced the expression of the inflammatory mediators TGF-β, MMP-1, and CXCR4, which are involved in cancer cell proliferation (TGF-β), migration and metastasis (MMP-1 and CXCR4), and angiogenesis (MMP-1). [6], [8]–[10] Therefore, IL-19 directly affects tumor proliferation, migration, and progression and also indirectly affects such activities through inducing other mediators. We demonstrate that IL-19 is an important local mediator in the microenvironment that affects esophageal cancer cells progression.

A variety of direct cell-cell, cell-matrix, and paracrine interactions are involved in metastasis. MMP is involved in endothelial cell injury when tumor cells cross the endothelial barrier [43]. Cytokines are intercellular mediators that regulate survival, growth, differentiation, and the effector functions of cells [44], [45]. They also represent a network with a large variety of different members that may promote tumor growth. Thus, we believe that IL-19 produced by esophageal cancer cells promotes tumor progression through its autocrine effect and by providing a microenvironment for tumor progression. Furthermore, cytokines are mediators of the effector response from innate and acquired cellular immunities [11], [12]; they are probably involved in the mechanism for tumor cell evasion from the immunosurveillance system. This might also be one of the mechanisms by which overexpression of IL-19 in esophageal cancer cells promotes tumor progression *in vivo*.

Several studies have shown that esophageal cancer is associated with an inflammatory environment. Takahashi *et al.* [46] reported that TGF-β was upregulated in esophageal cancer patients. Others [40] have reported increased proliferation in esophageal cancer through P-38 and ERK-MAPK pathways. The activation of intracellular signaling NF-κB promotes transcriptional upregulation of a wide variety of genes that are related to inflammation and tumor growth which is also important in esophageal cancer [47]. Our results in this study showed that IL-19 induced TGF-β, phosphorylated, P-38, Jnk, Erk1/2, Akt, and NF-κB in CE81T cells. We hypothesize that IL-19 induces NF-κB phosphorylation, then induces TGF-β and cyclin B1 gene upregulation, and finally induces P-38, Jnk, Erk1/2, and Akt phosphorylation.

The prognosis of esophageal cancer is generally poor because of extensive local invasion and frequent lymph node metastasis [5], [48]. We found that the colony forming levels were significantly higher in IL-19-treated CE81T cells. IL-19 also upregulates genes related to invasion, such as MMP-1, which degrade ECM. The association between the IL-19-induced increase in colony formation with MMP-1 in CE81T cell lines needs additional investigation. We did not show the clinical follow up for patient survival because our patient data recruited were within 2 years that is not long enough for survival analysis. However, we did find the clinicopathological variables correlated to IL-19 expression; high IL-19 expression associated with advanced tumor stages. More clinical follow up analysis in the future could provide further evidence of IL-19 expression in clinical outcome.

In addition to local invasion, the poor prognosis of esophageal cancer is also caused by lymph node metastasis [5], [48]. Sasaki K *et al.* (16) reported that esophageal squamous cell carcinoma expressed CXCR4, and Li *et al.* [49] that CXCR4 is related to cancer metastasis. Other studies [50]–[54] showed that metastasis has an organ-specific feature and that cytokines are involved in organ-specific metastasis. We previously reported (32) that in breast cancer IL-19 induces fibronectin expression and assembly, which is associated with lung metastasis. [32] In the present study, IL-19 was not involved in fibronectin assembly in CE81T cells (data not shown). Nevertheless, IL-19 promoted migration and induced CXCR4 expression in CE81T cells; anti-IL-19 mAb inhibited that expression, which indicated that the effects of IL-19 were specific. The measurement of IL-19 on *in vivo* metastasis in a mouse model remains to be determined. Our *in vivo* experiment showed that anti-IL-19 monoclonal antibody inhibited CE81T tumor growth. The expression of IL-19, TGF-β, MMP-1, CXCR4, and cyclin B1 in the tumors was also downregulated by 1BB1 treatment. We hypothesize that IL-19 augments cancer progression through its autocrine signaling, and that IL-19 may be the target for therapeutic in esophageal cancer.

IL-19 activates the type I IL-20 receptor complex (IL-20R1/IL-20R2), which is also activated by IL-20 and -24. Although IL-24 was reported (55) to be a tumor suppressor, the biological functions of IL-19 and IL-24 are different. Additional investigation of the activation of the type I IL-20 receptor complex by both IL-19 and IL-24 and the subsequent intracellular responses in tumor cells is needed.

We conclude that IL-19 has an autocrine effect and provides a microenvironment that affects tumor progression in esophageal cancer. Antagonizing IL-19 may have therapeutic potential in esophageal cancer.
